# Facile and scalable synthesis of sub-micrometer electrolyte particles for solid acid fuel cells†

**DOI:** 10.1039/c8ra03293a

**Published:** 2018-06-13

**Authors:** F. P. Lohmann-Richters, C. Odenwald, G. Kickelbick, B. Abel, Á. Varga

**Affiliations:** Leibniz Institute of Surface Engineering (IOM) Permoserstr. 15 D-04318 Leipzig Germany aron.varga@iom-leipzig.de +49 341 2353364; Saarland University, Inorganic Solid State Chemistry, Campus, Building C4 1 66123 Saarbrücken Germany

## Abstract

Nanostructuring fuel cell electrodes is a viable pathway to reach high performance with low catalyst loadings. Thus, in solid acid fuel cells, small CsH_2_PO_4_ electrolyte particles are needed for the composite powder electrodes as well as for thin electrolyte membranes. Previous efforts have resulted in significant improvements in performance when using sub-micrometer CsH_2_PO_4_ particles, but laborious methods with low throughput were employed for their synthesis. In this work, we present a simple, robust, and scalable method to synthesize CsH_2_PO_4_ particles with diameters down to below 200 nm. The method involves precipitating CsH_2_PO_4_ by mixing precursor solutions in alcohol in the presence of a dispersing additive. The influence of the concentrations, the batch size, the solvent, and the mixing process is investigated. The particle size decreases down to 119 nm with increasing amount of dispersing additive. Mixing in a microreactor leads to a narrower particle size distribution. The particle shape can be tuned by varying the solvent. The ionic conductivity under solid acid fuel cell conditions is 2.0 × 10^−2^ S cm^−1^ and thus close to that of CsH_2_PO_4_ without dispersing additive.

## Abstract 2

Stable sub-micrometer CsH_2_PO_4_ electrolyte particles for application in solid acid fuel cells are precipitated in a facile, scalable way.

## Graphical abstract

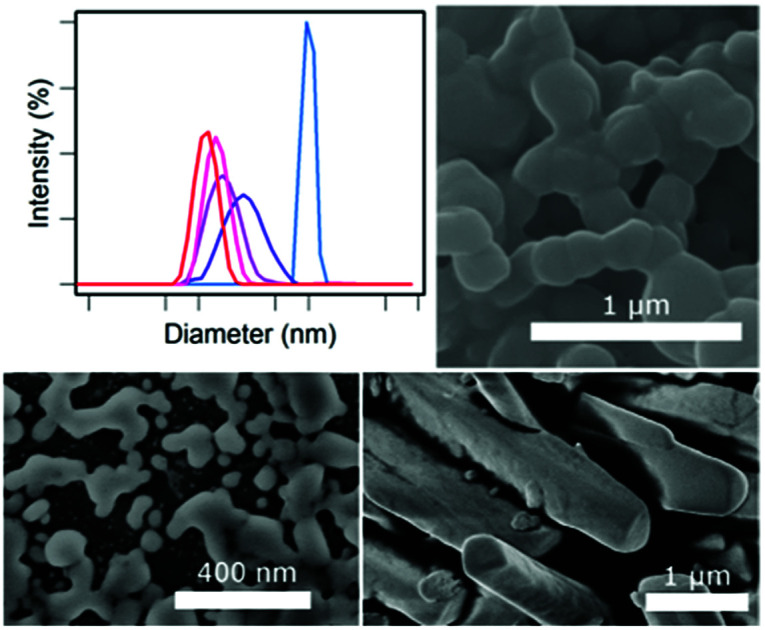

## 1 Introduction

The design of electrodes with a large electrocatalytically active surface area is crucial for most fuel cells. This is particularly challenging for fuel cells with solid electrolytes. Here, not only the electrocatalyst with its conductive support has to be optimized, but also the mixing with the electrolyte particles.

Solid acid fuel cells (SAFC) are usually based on CsH_2_PO_4_ as electrolyte. At the operating temperature of *ca.* 240 °C the electrolyte is in the superprotonic phase with high proton conductivity.^1,2^ The solid-state electrolyte and the intermediate operating temperature lead to a number of advantages, such as resistance to catalyst poisoning, cost-effective engineering materials, ease of handling, and simple water management.^3^ Nevertheless, the loading of platinum as electrocatalyst in SAFCs still has to be reduced to allow broad application. Small electrolyte particles can improve the performance of both the membrane and the electrodes in SAFCs.

SAFCs with electrolyte membranes as thin as 25 μm and a power density of 415 mW cm^−2^ have been reported in literature. However, the membranes were mechanically unstable and the cells showed a rapid decrease of power output.^4^ Composites of CsH_2_PO_4_ and inorganic oxides^3,5,6^ or polymers^7–9^ revealed improved mechanical stability and are promising for creating stable, thin electrolyte membranes. In any case, the membrane thickness is limited by the size of the electrolyte particles. To form a dense, non gas-permeable layer, a thickness of at least several particle diameters is necessary.

The electrode of state-of-the-art SAFCs consists of a mixture of electrolyte particles and platinum as electrocatalyst. The platinum utilization was significantly improved by using carbon nanotubes (CNT) as catalyst support.^10–12^ For electrodes in which the catalyst is deposited directly on the electrolyte particles, the power output was significantly increased by reducing the size of the electrolyte particles in the range of 100 to 200 nm.^3^ Here, the surface increase directly translates to an increase of the electrocatalytically active surface area (ECSA). Small electrolyte particles are therefore advantageous for high-performance electrodes and electrolyte membranes in SAFCs. The particle size is probably limited by the particle stability toward sintering or coalescence.^17^ It appears that an optimal size has not yet been determined. Only recently, a method to measure the ECSA in SAFCs *in situ* has been put forward.^13^

The conventional method to prepare CsH_2_PO_4_ is by precipitation from an aqueous solution through the addition of methanol,^2^ yielding particles in the range of several micrometers. The particle size can be reduced to about 1 μm using ball milling.^14^ Particles down to 0.5 μm were obtained by injecting an aqueous solution of CsH_2_PO_4_ through a fine needle into methanol under ultrasonication. Larger particles had to be removed by centrifugation, resulting in low yields.^15^

Even smaller particle diameters of 0.4 μm and 0.1 μm were achieved by spray drying^16^ and electrospray deposition,^17^ respectively. Both techniques resulted in improved platinum utilization (the inverse of the electrode impedance, divided by the platinum loading) when the particles were stabilized by the addition of polyvinylpyrrolidone (PVP) of high molecular weight. Unfortunately, the throughput of these techniques is insufficient for production-scale synthesis at this time.

The use of surfactants to synthesize nanoparticles of controlled size is well known and established for a large variety of materials. Hosseini *et al.* applied this approach for the precipitation of CsH_2_PO_4_.^18^ The authors reported particle sizes of approximately 10 nm in diameter. The amount of surfactant necessary to achieve such a small size was very high, 62 wt% in relation to CsH_2_PO_4_. Unfortunately, the stability and agglomeration of the particles under SAFC operating conditions was not investigated. Even significantly larger particles from electrospray deposition needed to be stabilized by the addition of PVP, and the melting point of the surfactant employed here is near the operating temperature of SAFCs. It is therefore unlikely that a stable electrode performance can be obtained.

For SAFC electrodes, it can be beneficial to employ electrolyte particles which are not only small, but also have a high aspect ratio, such as rods or platelets. These shapes facilitate the percolation of the electrolyte in a porous powder electrode. Ahn *et al.* have shown that the shape and size of CsH_2_PO_4_ particles can be influenced by the addition of ethylene glycoles and acetonitrile in the precipitation.^19^

The supersaturation of the solution is one of the most important factors determining the size of precipitated particles.^20^ Apart from the concentration of the involved solutions, it depends heavily on the mixing process. An extremely fast and very well controlled way to mix two liquids is the use of micromixers or -reactors. Microreactors are generally three-dimensional devices with inner dimensions in the range of 1–1000 μm.^21^ Above all, microreactors offer rapid mixing times, typically in the order of milliseconds. This improves the mass transport and leads to homogeneous conditions that are ideal for the particle formation in fast precipitation reactions. The constant operating conditions lead to better process control, higher reproducibility, and improved yield, compared to batch methods.^22,23^ The suitability of the microjet reactor method^24^ for different precipitation reactions was already demonstrated, *e.g.* for barium sulfate,^25^ titanium dioxide,^26^ zinc oxide, brushite and magnetite particles,^27^ and for the sol–gel synthesis of ORMOSIL microspheres.^28,29^

The precipitation of BaSO_4_ is a common model reaction for studying the formation and agglomeration of nanoparticles.^20,25,30,31^ Often, dispersing additives are employed in order to stabilize the nanoparticle suspensions. To our knowledge, for the precipitation of CsH_2_PO_4_ neither the use of microreactors or other continuous mixing devices, nor the addition of dispersing additives have been reported in literature.

In this work, we present the simple and scalable precipitation process yielding sub-micrometer CsH_2_PO_4_ particles for the application in SAFCs. This is achieved by the addition of a commercially available dispersing additive. The influence of the concentrations, the solvent, and the mixing procedure is investigated.

## 2 Materials and methods

MelPers 0045, identified as a promising dispersing additive in preliminary tests, was kindly provided by BASF Construction Solutions. It is a *ca.* 45 wt% aqueous solution of polycarboxylate ether comb copolymer with a charged backbone, providing electrostatic as well as steric stabilization. MelPers 0045 is usually employed to disperse inorganic pigments.^32^ CsOH hydrate (99.9% metal basis, abcr; est. water content 12.5%) was used as Cs^+^-source instead of the widely used Cs_2_CO_3_ to avoid carbonate residues and gas formation, especially in the microreactor.

### 2.1 Batch precipitation

The synthesis of CsH_2_PO_4_ particles *via* batch precipitation was typically performed as follows: H_3_PO_4_ (0.13 g, 1.09 mmol; 85%, Carl Roth) was mixed with EtOH (technical grade, Carl Roth) and MelPers 0045 (0.05 g, 20 wt% of the expected dry product) was added. The solution was diluted to 25 mL and a solution of CsOH (0.17 g, 1.09 mmol) in EtOH (25 mL) was added under intense stirring with a magnetic stir bar. The solution immediately turned white, indicating the formation of the product. This protocol with 20 wt% MelPers 0045, nominally 0.25 g of CsH_2_PO_4_ with a nominal concentration of 5 g L^−1^ served as reference point for varying the parameters.

Different amounts of the dispersing additive, relative to the expected product, were used (0, 2, 5, 20, 40 wt%). Note, that the dispersing additive has a solid content of 45 wt%. The expected fractions of polymer in the dried products are therefore 0, 0.9, 2.3, 9.0 and 18.0 wt%. For comparison, CsH_2_PO_4_ was also prepared by the conventional precipitation method^1,2^ by adding EtOH to an aqueous solution, but with the addition of 20 wt% dispersing additive.

The batch size was scaled from 0.25 g over 1 g to 4 g, with all concentrations kept constant. From the latter, a part of the product was collected for further analysis by centrifugation (Sigma 3-18K) with a relative centrifugal force of 4248 for 30 min and dried at 120 °C under reduced pressure.

The nominal product concentration was varied from 1 g L^−1^ over 5, 10 and 40 g L^−1^ to 50 g L^−1^. For the latter, 35 mL of EtOH had to be employed to dissolve the CsOH, due to its limited solubility.

To investigate the influence of the solvent, the precipitation was also performed using MeOH (99%, Carl Roth), isopropanol (iPrOH, technical grade, Carl Roth), a 1 : 1 (vol) mixture of EtOH and acetonitrile (Uvasol quality, Merck) (denoted EtOH–ACN), and a 1 : 1 (vol) mixture of EtOH and ethylene glycol (99.8%, Sigma-Aldrich) (denoted EtOH–EtGly).

### 2.2 Microreactor precipitation

The influence of the mixing process was investigated by comparing the batch experiments with the precipitation in a microreactor.

A 125 mL solution of CsOH (1.02 g, 5.45 mmol; 99.9% metal basis, 15–20% H_2_O, alfa aesar) in EtOH (99%) was prepared. H_3_PO_4_ (0.55 g, 5.45 mmol; 98% p.a., Merck) with 5 and 20 wt% (0.06 g and 0.25 g) MelPers 0045 was diluted with 125 mL EtOH. The nominal resulting CsH_2_PO_4_ concentration was 5 g L^−1^.

The microreactor setup has been described in detail in a previous publication.^29^ In short, two HPLC pumps were used to press the precursor solutions through opposing horizontal nozzles of 300 μm diameter. The solutions collide as fine impinging jets in the middle of the chamber where a fast mixing in the order of 10 ms takes place. A nitrogen gas flow (8 bar) orthogonal to the feeding streams supports the transfer of the mixture to the collecting vessel. The reaction was performed at 20 °C with a flow rate of 250 mL min^−1^ for each precursor solution.

### 2.3 Characterization

The particle size distributions were measured with dynamic light scattering (DLS) using a Zetasizer Nano ZS (ZEN3600, Malvern Instruments, 173° scattering angle) using the as-precipitated suspensions. The measurements were performed at 20 °C with an equilibration time of 2 min. No significant particle concentration in EtOH or the solution of the dispersing additive in EtOH was observed. The viscosity and the refractive index of the pure solvents was employed for the data analysis. The data is reported as intensity distributions as this is the quantity directly obtained from DLS. A conversion to the distribution by number or volume would be possible, but involves additional assumptions and requires detailed data on the particles' complex refractive index.

To confirm the particle size from DLS and to observe the morphology, scanning electron microscopy (SEM) images were recorded in a Carl Zeiss Ultra 55. For the microreactor samples, SEM images were recorded with a JEOL JSM-7000F after deposition of a gold layer to avoid charging effects.

The samples for SEM were prepared by drying a drop of the product suspension on a Si-wafer at 80 °C, or on a glass slide with subsequent transfer to a SEM sample holder. In order to evaluate the particles' agglomeration behavior, one SEM sample was exposed to SAFC operating conditions (240 °C, H_2_, *ca.* 0.4 atm H_2_O) for 13 h.

Using the product from the 4 g batch, X-ray diffraction (XRD) patterns were recorded on a Rigaku Ultima VI with Cu Kα1 radiation to confirm the phase. The crystallite size was calculated according to Scherrer from the full width at half maximum of the reflex at 29° assuming a shape factor of *K* = 1. Thermogravimetric analysis (TGA) (STA 449 C Jupiter, Netzsch) was performed with a heating rate of 10 °C min^−1^ under air to detect the additive's decomposition and the dehydration of CsH_2_PO_4_. Differential scanning calorimetry (DSC) (DSC 8500, PerkinElmer) was recorded with a heating rate of 10 °C min^−1^ to observe the dispersing additive's glass transition and the superprotonic phase transition of CsH_2_PO_4_.

For conductivity measurements under SAFC operating conditions, a 0.5 mm thick dense pellet with a radius of 1 cm was prepared by uniaxial pressing with 4 t for 1 min followed by 8 t for 5 min (Atlas Auto 15T, Specac Ltd.). The pellet was sandwiched between discs of carbon paper (TP-060, Toray) followed by stainless steel gas diffusion layers (PACOPOR ST 60 AL3, Paul GmbH und Co. KG) and heated to 240 °C under argon with 0.4 atm H_2_O. Impedance spectra (10 mV amplitude, 1 MHz to 200 mHz) were recorded with a VSP-300 potentiostat (Bio-Logic Science Instruments).

## 3 Results and discussion

### 3.1 Batch precipitation

The conventional synthesis of CsH_2_PO_4_ for SAFCs is based on the precipitation from an aqueous solution of the precursors by the addition of a non-solvent, usually methanol.^1,2^ The addition of the methanol gradually decreases the solubility of CsH_2_PO_4_, leading to precipitation. While the precursors are soluble in alcohols, the product is not. Thus, the precipitation can also be induced by mixing separate solutions of the precursors in alcohol, as in the new synthetic procedure presented in this work. The lower water content can be expected to reduce the agglomeration of the CsH_2_PO_4_ particles. A dispersing additive can be added to reduce the agglomeration further.

#### 3.1.1 Dispersing additive quantity

The precipitation of CsH_2_PO_4_ was performed with different amounts of the dispersing additive MelPers 0045. The obtained suspensions varied in their stability. In the sample without dispersing additive, complete sedimentation occurred within *ca.* 10 min. The suspensions with 2 wt% to 20 wt% of dispersing additive exhibited first indications of sedimentation between 10 min and several hours, increasing with the amount of MelPers. However, the sedimentation was not complete: even after several days the supernatant remained turbid. With 40 wt% of MelPers 0045, slight sedimentation was only observed after one week. After the partial sedimentation, the particles in all samples could be easily redispersed by vigorous shaking. This was even the case several months after the synthesis.

The sedimentation behavior of the sample conventionally precipitated from aqueous solution was similar to the above-mentioned suspension with 2 wt% dispersing additive, although it contained 20 wt% MelPers. However, the particles could not be redispersed after sedimentation, even with prolonged ultrasonication. Most probably, the much higher water content leads to coalescence of the sedimented particles. For stable CsH_2_PO_4_ nanoparticles it might thus be beneficial to have a low water content in the suspension. The amount of water in the samples precipitated from ethanol could be further reduced by employing reactants and solvents with low water content.

**Fig. 1:**
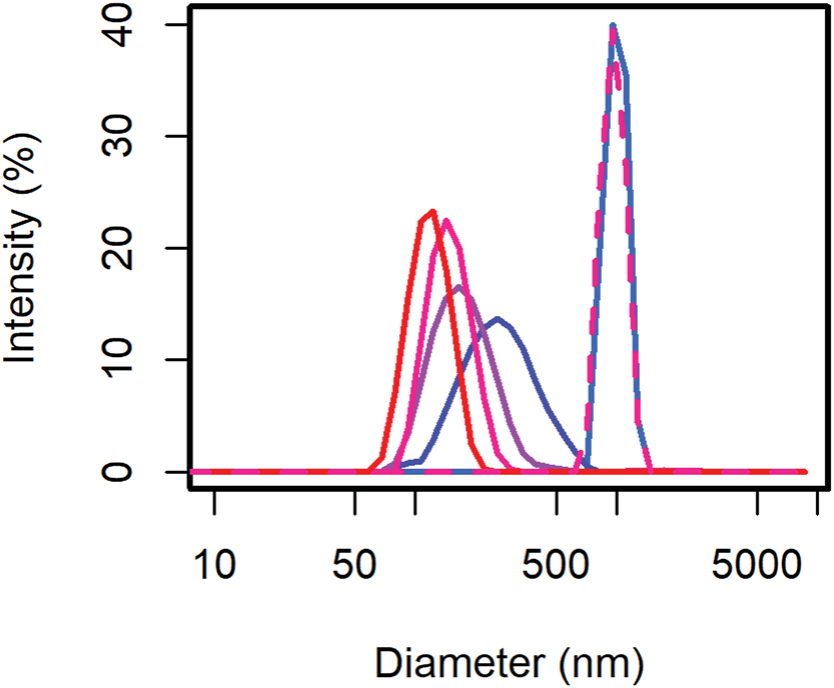
The CsH_2_PO_4_ particle size as measured with DLS decreases with the dispersing additive content of 0 (blue) to 40 wt% (red). Even with 20 wt%, the conventional precipitation from aqueous solution yields large particles (dashed).

From the scattering intensity distribution in DLS (Fig. 1), it can be seen, that the particle sizes decrease with increasing content of MelPers 0045. As the scattering intensity depends on the sixth power of the particle diameter, larger particles are over-represented in these intensity distributions. However, this distribution is the one directly obtained from the measurement, and any conversion involves additional assumptions. From 0 to 2 wt% dispersing additive, the maximum of the scattering intensity shifts from 998 nm to 278 nm. Between 2 wt% and 40 wt% the particle size decreases further and the distribution gets narrower (Table 1). With 40 wt% the distribution extends clearly to below 100 nm. The sample precipitated from aqueous solution with 20 wt% yields a particle size distribution similar to the 0 wt% sample, illustrating the benefit of the precipitation protocol presented in this work.

**Table 1:** Intensity maxima and standard deviations (SD) from DLS depending on the amount of dispersing additive (DA)

DA (wt%)	Intensity maximum (nm)	SD (nm)
0	998	121
2	278	117
5	178	64
20	150	37
40	119	27

**Fig. 2:**
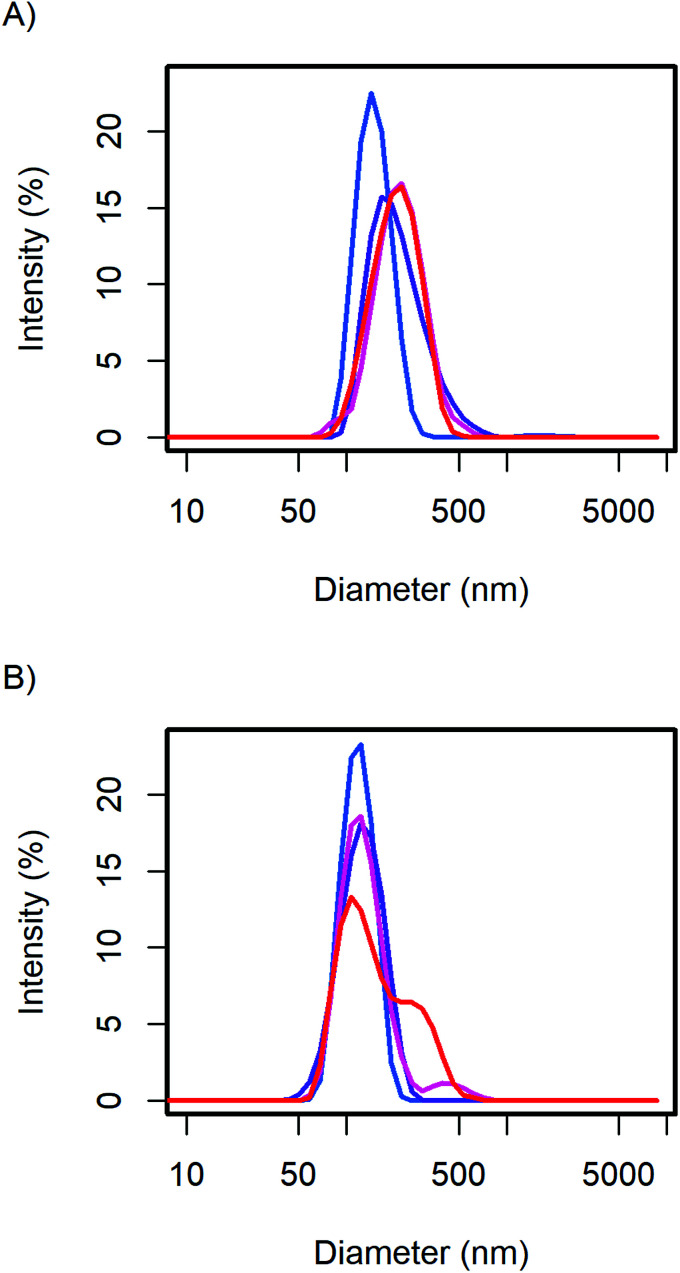
The CsH_2_PO_4_ particle size distributions of (A) the 20 wt% and (B) 40 wt% additive samples show minor changes over time from the first measurement (blue), after 1, 3, and 7 days (red).

The particle size distribution of the samples with 20 and 40 wt% MelPers 0045 was measured again after 1, 3, and 7 days (Fig. 2). With 20 wt%, the size distribution stabilized at slightly increased diameters. With 40 wt%, the size distribution developed a shoulder in the same diameter range in which the 20 wt% sample stabilized. The higher water content in the sample with higher additive concentration can be expected to play a role in the aging of the particles. However, both particle size distributions are remarkably stable, leaving more than enough time for further processing and analysis.

**Fig. 3:**
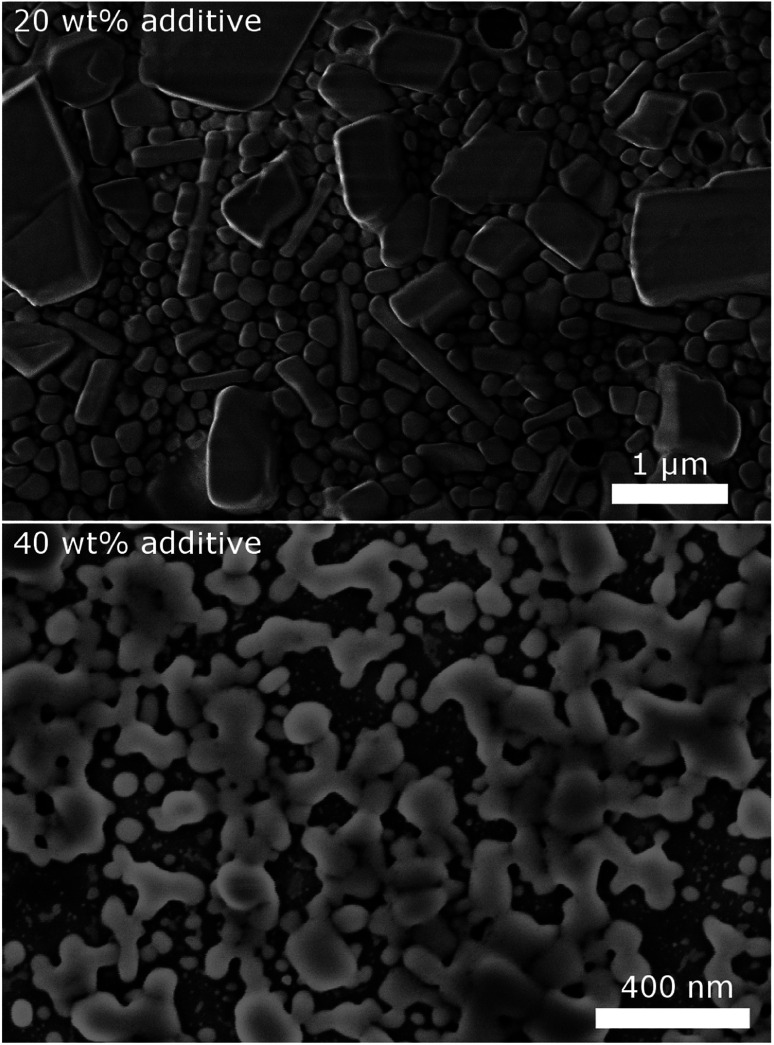
SEM images of CsH_2_PO_4_ particles show a decrease of particles size and changes in the shape with increasing amount of dispersing additive.

To validate the particle size measured by DLS, SEM images of selected samples were recorded (Fig. 3). Due to severe drying artifacts that can occur during the preparation of the SEM samples, the images can only serve for qualitative analysis. In accordance with DLS, many particles in the range of *ca.* 200 nm are observed with 20 wt% dispersing additive. In addition to small spherical particles, also rod-like particles and larger particles in the micrometer range are present. Such particles and large rods are also observed in the samples with lower content of dispersing additive. The larger particles might not be accounted for in the DLS measurements, as they sediment too fast. With 40 wt% of MelPers 0045, SEM reveals particles below 100 nm. This is in good agreement with the DLS data. Note, that neither large nor rod-shaped particles are observed. We presume that at this concentration the dispersing additive covers the particles' surface fast and completely, suppressing the growth of particles with increased size or aspect ratio. It is not yet clear, whether the rod-shape of some particles observed at lower additive concentrations is induced by the solvent, by the dispersing additive, by impurities in either, or a combination of these factors.

Despite the different measurement principles and different sources of error, the particle sizes observed in DLS and in SEM show good agreement. The determined particle sizes are therefore reliable. We note, that a dispersing additive content of 20 wt% is sufficient to yield particles of *ca.* 200 nm in diameter. The particle size shows no drastic changes between 5 and 40 wt%. The precipitation with 20 wt% MelPers 0045 is therefore employed in all following experiments.

#### 3.1.2 Batch size

**Fig. 4:**
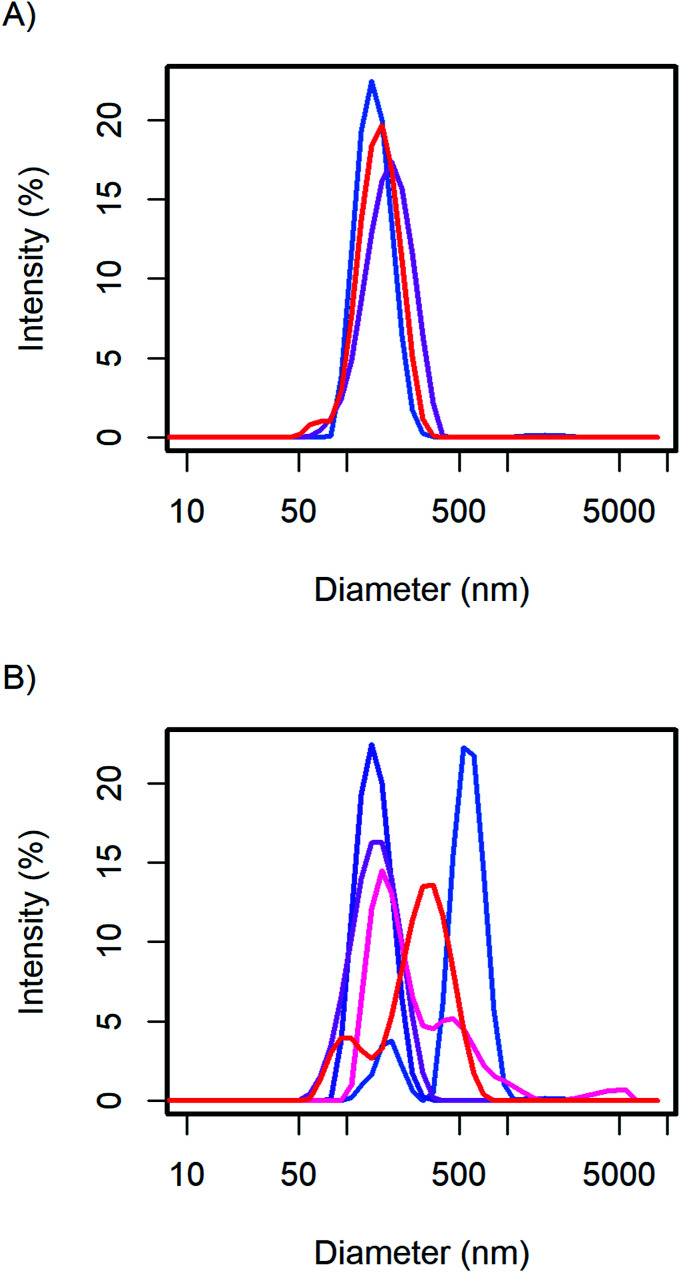
The CsH_2_PO_4_ particle size as measured with DLS shows (A) no clear trend when scaling the synthesis (0.25 (blue), 1, and 4 g (red)) but (B) changes with the nominal product concentration: 1 (blue), 5, 10, 40, and 50 g L^−1^ (red).

The change of the CsH_2_PO_4_ particle size with the batch size of the experiment with constant concentrations is presented in Fig. 4A. A slight variation of the particle size can be observed, but no trend is evident. Small differences in the mixing process in the beaker when pouring the precursor solutions together might be the origin of this variation.

From the 4 g batch size, the product was separated by centrifugation. Even after prolonged centrifugation not all particles had sedimented. A DLS measurement of the supernatant resulted in practically the same particle size distribution as the sample with 40 wt%. The smallest particles formed in the precipitation in the presence of the dispersing additive in a beaker are thus of a similar size, independent of its concentration. The reason might be that the microscopic mixing in the precipitation in a beaker is not well controlled. However, due to the strong size-dependence of the scattering intensity the smaller particles are not always evident in DLS.

#### 3.1.3 Nominal product concentration

The concentration of the solutions in the precipitation reaction influences the supersaturation and thus the particle size. Additionally, high concentrations are desired for large-scale synthesis. Thus the concentration dependence of the particle size was investigated.

We observe, that the samples with very low and very high concentration show faster sedimentation. This correlates very well with the particle size distribution observed in DLS (Fig. 4B). The suspensions with 1, 40, and 50 g L^−1^ show bimodal distributions: one part of the particles is in the range of 200 nm and thus similar to the particle size with 5 and 10 g L^−1^. A second part of the particles has significantly larger diameters.

At 1 g L^−1^ the larger particles might form because the lower concentration leads to a lower supersaturation and thus less nucleation. The lower concentration of the dispersing additive in the solvent could also lead to a less adsorption of the additive onto the surface, reducing its stabilizing effect. At elevated concentrations, the viscosity of the solutions probably increases, leading to slower mixing and thus again less supersaturation and fewer crystal nuclei. In addition, agglomeration should be more severe at higher concentration as more particle collisions occur. In the range of 5–10 g L^−1^ the size distribution shows only one peak around 200 nm. Here, the above-mentioned effects are not inhibiting the formation of stabilized small particles.

#### 3.1.4 Shape control

**Fig. 5:**
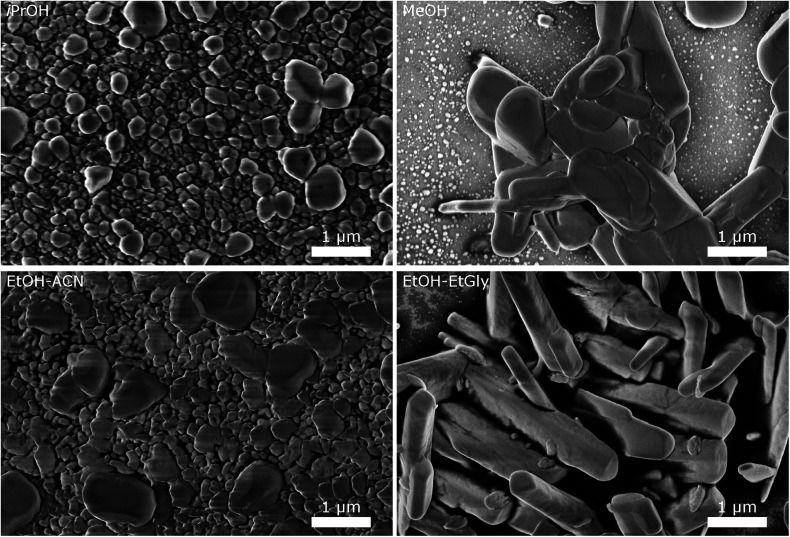
SEM images of CsH_2_PO_4_ particles precipitated in different solvents illustrate the influence of the solvent on the particle size and morphology.

It has been reported, that the solvent can influence the shape of the precipitated CsH_2_PO_4_ particles.^19^ In Fig. 5 SEM images of particles precipitated from various solvents are presented, with all concentrations as in the reference experiment with 5 g L^−1^ and 20 wt% MelPers 0045. The DLS particle size distributions of sufficiently stable suspensions are given in the ESI (Fig. S1†).

The particles from EtOH are mostly spherical, but with a significant number of rod-shaped particles and some larger ones, as observed in the experiments described above. Isopropanol yields spherical particles with a broader size distribution and no rods are observed. In MeOH, we observe a tendency for larger particles, although a significant number of small particles below 200 nm is also present. The mixture of EtOH with acetonitrile (EtOH–ACN) yielded mostly spherical particles, as in EtOH. While acetonitrile has been reported to lead to rod-shaped particles,^19^ we do not observe this effect with our precipitation protocol.

With a mixture of EtOH and ethylene glycol (EtOH–EtGly), no precipitation was observed when mixing the precursor solutions. Only on the next day, fine needles could be recognized. SEM revealed the presence of rod-shaped particles of slightly larger dimensions than in the precipitation with pure EtOH. This agrees with the finding of Ahn *et al.*, who observed the formation of rods in a mixture of MeOH and ethylene glycol.^19^ In addition, more than 3 μm long needles were observed. However, these probably formed while drying the SEM sample.

These results show, that the particle morphology and size can be influenced by the choice of the solvent, also in presence of the dispersing additive. While small spherical particles are good for producing thin layers as electrolyte membranes for SAFCs, particles with a large aspect ratio are beneficial for producing porous electrodes. Further investigations with different solvents and solvent mixtures, should allow the precipitation of such particles. Our results with ethanol and ethylene glycol are already very promising. Good control of the mixing process should be essential for homogeneous particle shapes.

### 3.2 Microreactor precipitation

**Fig. 6:**
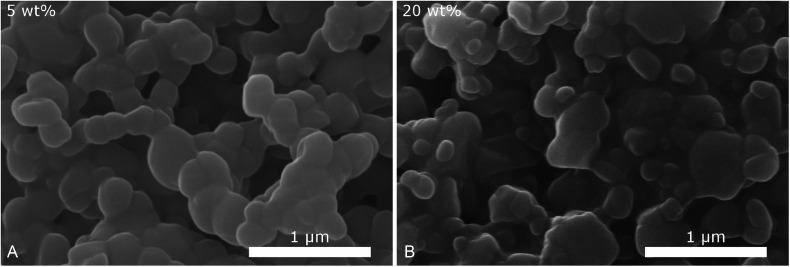
SEM images of CsH_2_PO_4_ particles produced with the microjet mixer with (A) 5 wt% and (B) 20 wt% of dispersing additive show similar sizes but narrower size distribution than precipitation in a beaker.

To determine, how the mixing process and its time scale influence the particle size, we conducted the precipitation in a microreactor with an extremely short mixing time (*ca.* 10 ms (ref. 29)), compared to the conventional experiments in simple glass beakers. In Fig. 6 SEM images of the particles obtained with the same concentrations as the standard 5 g L^−1^ precipitation with 5 and 20 wt% of dispersing additive are given. Although the particles partly agglomerated during the drying process, a decrease in size can be observed with increasing concentration of dispersing additive. For both samples the particles have similar sizes, below 200 nm, as those precipitated by mixing in a beaker. However, in contrast to these, no significantly larger particles are observed. This indicates that as expected, the continuous, thorough mixing in the microreactor yields a narrower particle size distribution than the rather uncontrolled mixing in a beaker.

For an otimized precipitation of BaSO_4_ in a microreactor particles down to 60 nm have been reported.^25^ The authors mentioned that although the primary particles and the crystallite size from XRD were in the order of 25 nm, increasing the amount of dispersing additive did not yield smaller particles. The particle size was mainly controlled by agglomeration and not by crystal growth.

We analyzed the crystallite size of selected samples from the batch precipitation by XRD: the crystallite size of the 4 g batch product was 42 nm. XRD of the sample without dispersing additive yielded 44 nm, the conventional precipitation from aqueous solution without dispersing additive 41 nm. Keeping in mind the limitations of the Scherrer equation, the differences are not significant. The particle size is thus not determined by the crystallite size but by the degree of agglomeration, as in the precipitation of BaSO_4_. It can therefore be expected, that the particle size can still be slightly reduced employing the microreactor with a high concentration of dispersing additive. However, the study on BaSO_4_ indicates that the diameter will probably remain above two times the crystallite size.

### 3.3 Application in SAFCs

For the application of the CsH_2_PO_4_ particles in SAFCs, their conductivity and stability under the operating conditions are important. Therefore, further analysis was performed with the product of the 4 g batch. The X-ray diffractogram (ESI Fig. S2†) shows the same pattern as pure CsH_2_PO_4_. Slight variations in the intensity of the reflexes probably arise from different preferred orientations of the crystallites.

**Fig. 7:**
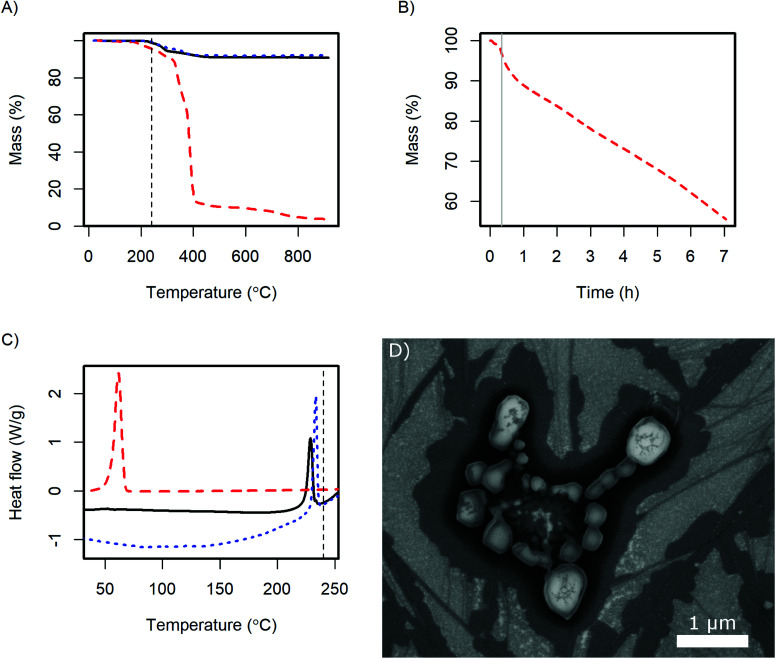
TGA (A) shows the thermal stability of the CsH_2_PO_4_ particles (black line) and its constituents, CsH_2_PO_4_ (dotted blue line) and MelPers 0045 (dashed red line) under air, and (B) the weight change of MelPers 0045 at 240 °C after a short ramp to this temperature (grey line). In DSC (C), the glass transition of MelPers 0045 as well as the superprotonic transition of CsH_2_PO_4_ is observed for the CsH_2_PO_4_ particles. The SAFC operating temperature of *ca.* 240 °C is indicated by a vertical dashed line. (D) SEM image of CsH_2_PO_4_ particles after exposure to SAFC operating conditions (240 °C, H_2_, 0.4 atm H_2_O) for 13 h.

In DSC the glass transition of the polymer as well as the superprotonic transition of CsH_2_PO_4_ are observed as in the pure compounds (Fig. 7C). The content of the dispersing additive in the dry product is nominally 9 wt% and the glass transition probably changes due to the interaction with the CsH_2_PO_4_. Therefore the glass transition peak in the product is very small. The observed enthalpy of the superprotonic transition of the particles is 10 kJ mol^−1^. Given, that the nominal content of CsH_2_PO_4_ is 91%, this is in good agreement with the literature value of 11.7 ± 1.1 kJ mol^−1^.^33^

TGA reveals, that the dispersing additive exhibits minor weight loss starting at 170 °C and a very rapid decrease above 320 °C (Fig. 7A). Held at the SAFC operating temperature at 240 °C, a continuous weight loss is observed (Fig. 7B). The additive can therefore be removed by thermal decomposition, which should allow the use of high quantities of the dispersing additive in the precipitation without diminishing the proton conductivity under SAFC conditions.

The weight loss of CsH_2_PO_4_ precipitated with MelPers 0045 is essentially a superposition of the degradation of the dispersing additive and the well-known dehydration of the salt.^1^ Unfortunately, these two processes overlap and might slightly differ from the pure compounds. As the dispersing additive is decomposed to a large extent at high temperatures, calculating its mass fraction in the product is prone to error. Nevertheless, with the weight loss at the plateau at 550 °C we calculate a fraction of 9.5 wt%. This is in good agreement with the amount of employed dispersing additive, which was 20 wt% of the 45 wt% solution of the dispersing additive.

As the crystal structure of the CsH_2_PO_4_ particles corresponds to bulk CsH_2_PO_4_ and the superprotonic transition is observed in DSC, proton conduction can be expected. The conductivity of a 0.5 mm thick pellet under SAFC operating conditions increased to 2.0 10^−2^ S cm^−1^ within 4 h. The impedance spectrum is given in Fig. S3.† The observed conductivity is slightly lower than the value of 2.2 × 10^−2^ S cm^−1^ reported for pure CsH_2_PO_4_.^1^ This difference can be assigned to the dispersing additive or its decomposition products at the particle interfaces. However, the conductivity kept increasing toward the literature value with the slope depending on the gas flow at the electrodes. The dependence on time and gas flow probably reflects the continuous decomposition of the additive, which can be expected to proceed more slowly in the dense pellet than in the loose powder in TGA under air.

The stability of the particles with respect to sintering was evaluated by exposing SEM samples to SAFC operating conditions, Fig. 7D. It can be seen that the particles sinter but do not coalesce completely. So although the additive is not stable at 240 °C, the electrolyte particles are surprisingly stable. The long-term stability of a porous electrode fabricated from these particles needs further investigation. It might be necessary to add a temperature-resistant polymer like PVP to stabilize the structure, see ref. 16 and 17. However, such polymers also have a negative effect on the ion conductivity in the electrodes, which will be discussed in a different publication. For preparing thin electrolyte membranes, sintering is not an issue as a dense layer is desired.

**Fig. 8:**
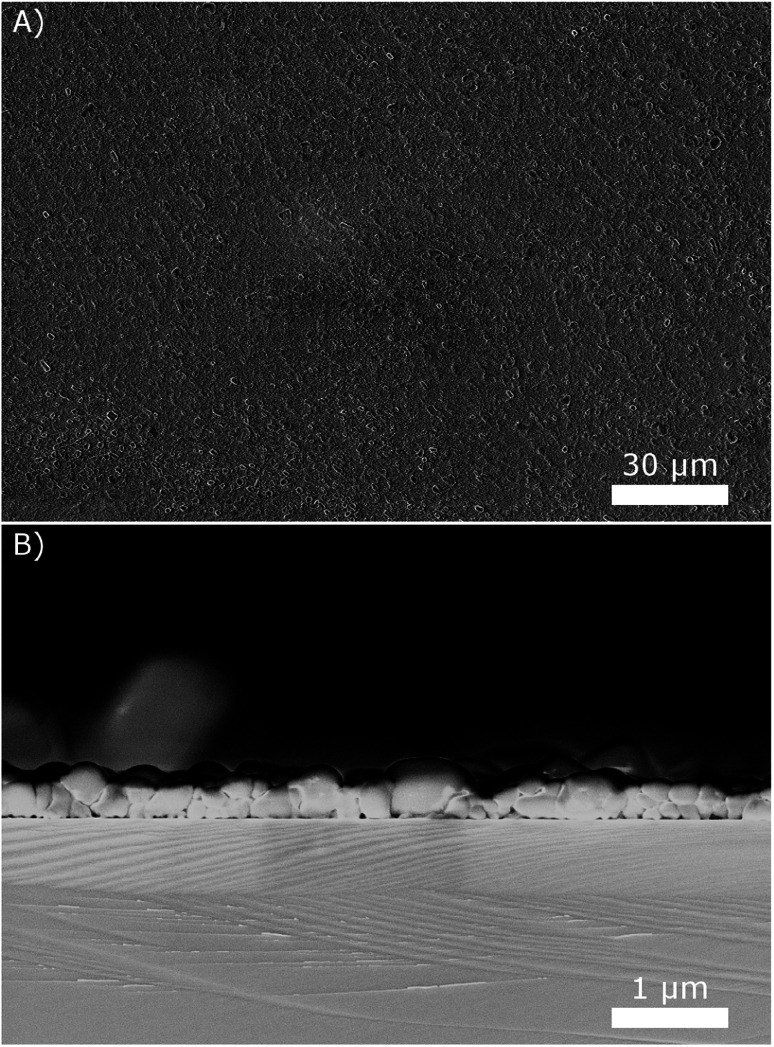
(A) SEM images of CsH_2_PO_4_ particles precipitated with 10 g L^−1^ and drop coated on a wafer show good coverage of the surface. (B) The side view of the sample broken in half reveals the thickness of the layer.

The next step is the development of fabrication processes for SAFC electrolyte membranes and electrodes from the obtained suspensions. As for thin electrolyte membranes, we observed that drop coating the suspensions on silicon wafers at 80 °C results in quite uniform thin layers with good surface coverage. An example is given in Fig. 8, using the 10 g L^−1^ suspension. The side view of the layer reveals that it is only a few particles thick, resulting in a total thickness below 1 μm. Repeating the coating several times resulted in correspondingly thicker layers.

## 4 Outlook and conclusion

In this work, we presented a simple, robust, and scalable method to prepare CsH_2_PO_4_ particles down to below 200 nm in diameter for application in solid acid fuel cell electrodes and electrolyte membranes. Stable suspensions of CsH_2_PO_4_ particles were obtained by mixing solutions of CsOH and H_3_PO_4_ in alcohols in the presence of the commercially available dispersing additive MelPers 0045. We investigated the influence of the concentrations, the batch size, the solvents, and the mixing process and found that the particle size and shape can be tuned. Compared to electrospray deposition and spray drying, which are the state-of-the art methods for synthesizing sub-micrometer CsH_2_PO_4_ particles, the presented precipitation is much simpler and at the same time yields similar particle sizes. The new method should be suitable for continuous production of small electrolyte particles for the application in SAFCs on a commercial scale.

The next step is to establish protocols for fabricating thin electrolyte membranes and porous electrodes from the suspensions. Our preliminary drop-coating results point out the potential for obtaining dense CsH_2_PO_4_ layers of only few micrometers in thickness. For application to larger areas, the stable suspensions could be applied with a spray gun as commonly used in painting. Independent of the mode of application, polymeric binders like PVDF or epoxi resin can be added to improve the mechanical properties of the layer.^7–9^ Another promising candidate would be polyamide-imides as these are thermally and mechanically stable, available in soluble form, and can be cured at temperatures around the operating temperature of SAFCs.

For the preparation of porous composite electrodes for SAFCs, the particles could for example be transferred to toluene and mixed with the supported electrocatalyst to form a slurry or suspension, which can be applied by spraying or screen printing. Here, the new sub-micrometer particles could be combined with recently reported platinum nanoparticles on CNTs to yield high-power SAFCs.^10,11^ First experiments adding CNTs before and after the precipitation as described in this work, indicate, that very intimate mixtures can be obtained, potentially maximizing the amount of active sites.

## Conflicts of interest

There are no conflicts of interest to declare

## Supplementary Material

RA-008-C8RA03293A-s001
