# Supplementary material for: Facile and scalable synthesis of sub-micrometer electrolyte particles for solid acid fuel cells
Source: RSC Adv. 2018 Jun 13;8(39):21806–15. doi: 10.1039/c8ra03293a (PMC9080985; doi:10.1039/c8ra03293a)
Supplement: RA-008-C8RA03293A-s001 [file RA-008-C8RA03293A-s001.pdf]

Electronic Supplementary Material (ESI) for RSC Advances.
This journal is © The Royal Society of Chemistry 2018

Supporting information

Facile and scalable synthesis of sub-micrometer electrolyte particles for solid acid fuel cells

F. P. Lohmann-Richters,[a] C. Odenwald,[b] G. Kickelbick,[b] B. Abel,[a] and Á. Varga*[a]

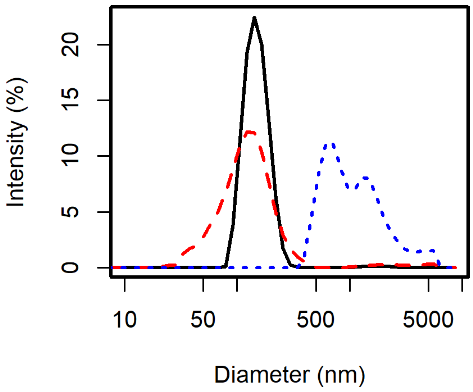

Figure S1: The CsH_2PO_4 particle size as measured with DLS depends on the choice of the solvent; EtOH (black), *i*PrOH (dashed, red), MeOH (dotted, blue).

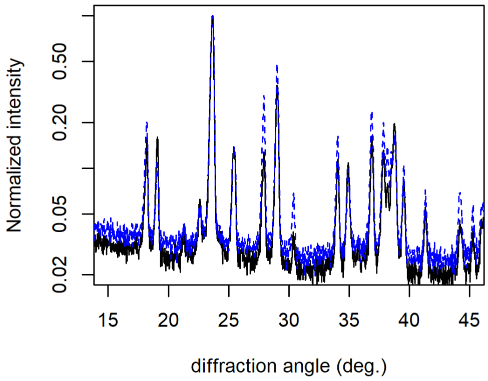

Figure S2: The X-ray diffractogram of CsH_2PO_4 prepared with 20 wt% dispersing additive (black) shows good agreement with that of pure CsH_2PO_4 (blue, dashed).

[a] *Leibniz Institute of Surface Engineering (IOM), Permoserstr. 15, D-04318 Leipzig, Germany. Tel: +49 341 2353364; E-mail: aron.varga@iom-leipzig.de*
[b] *Saarland University, Inorganic Solid State Chemistry, Campus Building C4 1, 66123 Saarbrücken, Germany.*

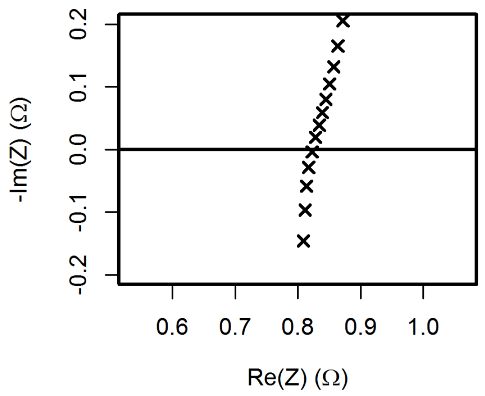

Figure S3: The electrochemical impedance spectrum of a 1 cm radius and 0.5 mm thick pellet of the material from the 4 g batch.
